# Modular Homes as a New Form of Accommodation to Tackle Homelessness: A Case Study From Cambridge, England

**DOI:** 10.1007/s10745-023-00404-1

**Published:** 2023-04-12

**Authors:** Richmond Juvenile Ehwi, Kwadwo Oti-Sarpong, Gemma Burgess, Johannes Lenhard, Eana Meng

**Affiliations:** 1 Department of Land Economy, University of Cambridge, Cambridge, UK; 2 Centre for Smart Infrastructure and Construction, Department of Engineering, University of Cambridge, Cambridge, UK; 3 Department of Social Anthropology, Max Planck Institute, University of Cambridge, Cambridge, UK; 4 Harvard Medical School, Harvard University, Boston, MA USA; 5 Centre for Housing and Planning Research, Department of Land Economy, 19 Silver Street, Cambridge, CB3 9EP USA

**Keywords:** Homelessness, Modular housing, Temporary accommodation, Social work, Wrap-around support services, Qualitative research, Cambridge, England

## Abstract

In England, provision of temporary accommodation for people experiencing homelessness has often entailed using traditional construction approaches to deliver housing. However, recent experiments are using modular homes to provide temporary accommodation, accompanied by support services for people experiencing homelessness. Given the early nature of these trials, it is unclear what impacts these modular homes have on their occupants and how these projects in turn impact surrounding residents and businesses. We present a case study of the first modular homes for people experiencing homelessness in Cambridge, England, drawing on longitudinal interviews with the six residents occupying these homes. We found that the physical features of the homes, coupled with wrap-around support services, yielded positive short- and mid-term outcomes for occupants, including improved management of their substance use and money, skills development and readiness for employment, social relations, and a burgeoning sense of community, safety, and security. These positive outcomes have spurred wider interest, including the incorporation of modular homes as alternative temporary accommodation in the Homelessness and Rough Sleeping Strategy of Cambridge City Council, alongside a growing research interest in modular homes and other new schemes by the national government. We argue for further empirical studies of the impacts of different modular home projects, including those that admit more diverse resident cohorts and offer different accommodation types to establish a clear methodology for future modular homes projects in England and beyond.

## Introduction

Homelessness is a widespread social problem across most developed countries, including the UK (Bullen & Baldry, 2019; Fitzpatrick et al., 2021). In England, for example, since the first autumn headcount of people sleeping rough, the most visible form of homelessness, their numbers rose from 1,768 to 2010 to a peak of 4,751 in 2017, before declining to 2,688 in 2020 (Ministry of Housing, Communities and Local Government (MHCLG), 2021a).

The UK government recognises homelessness as a social problem that will require support from central and local government and additional stakeholders, such as local businesses, communities, and faith-based groups, among others (MHCLG, 2018). In its 2018 ‘Rough Sleepers Strategy,’ the government committed to halving the number of people sleeping rough by 2022 and to ending homelessness altogether by 2027 (ibid.). Consequently, an array of initiatives and actions were put in place, including: the Homelessness Reduction Act 2017, assigning new homelessness prevention responsibilities to local authorities; the commitment of different funding packages for local homelessness-relief and prevention initiatives; delivery of more social housing, maintaining housing benefit for all supported housing, and ensuring secure, safe, and affordable homes in the private rental housing sector (MHCLG, 2018).

Traditionally, tackling homelessness in England and elsewhere relies heavily on the use of temporary forms of accommodation such as emergency night shelters, hostels, and bed and breakfast accommodation (Evans, 2020; Fitzpatrick et al., 2021). In England, 96,060 people lived in temporary accommodation as at the end of September 2021 (MHCLG, 2021a). This figure, according to the Homelessness Monitor, represents a 4% increase compared to the previous year (Crisis, 2022). Temporary accommodation options for people experiencing homelessness are often modelled around the ‘staircase approach’ that ‘entail(s) withholding permanent housing until homeless people demonstrate that they are housing ready’ (Clarke et al., 2020:0.954). There is an extensive literature on temporary accommodation, focusing among other things on different forms of temporary accommodation (Busch-Geertsema & Sahlin, 2007; Watts et al., 2018; Wilson & Barton, 2022), the types and nature of support services provided (Anderson, 2010; Clarke et al., 2020), and the lived experiences of different service users (Mitchell et al., 2004; Watts et al., 2018).

However, there is growing attention in the media and grey literature regarding the use of converted shipping containers and purpose-built prefabricated modular homes to provide housing for people experiencing homelessness. Exemplars in the form of apartments built from shipping containers, 3-D printed and prefabricated houses are being used to tackle homelessness in many cities, including Los Angeles in the USA, Chennai in India (Henry, 2021), Toronto and Scarborough in Canada (Adler, 2020), and Cambridge in England (Karampour & Burgess, 2022). In the UK, a nationwide mapping of this new approach by Karampour & Burgess (2022) notes that these schemes differ across: (1) construction methods (e.g., modular versus shipping containers); (2) scheme sizes (< 20 units versus ≥ 20 units on site); (3) household groups (e.g., families versus singles); and (4) site availability and length of planning permission conditions (e.g., ‘meanwhile’ sites waiting to be developed for other uses versus permanent sites).

However, so far, there has been no empirical evidence of the impact of these new schemes on people experiencing homelessness, and how these schemes in turn impact the wider society. Based on unique early access to participants in the first modular homes scheme (both residents and other stakeholders) in England, we explore the extent to which such schemes engender positive outcomes for people experiencing homelessness in terms of: (1) how the modular homes have improved the living conditions of the residents who occupy them; (2) what crucial issues and questions should be considered when using modular homes as an alternative form of temporary accommodation for people experiencing homelessness; and (3) how the scheme is impacting the environment beyond residents.

We first provide empirical evidence that can inform interventions by different stakeholders involved in tackling homelessness who may be contemplating similar schemes while contributing to the literature on existing approaches to tackling homelessness (Tsemberis et al., 2004; Bullen & Baldry, 2019). Secondly, we hope to contribute to current housing policy thinking about alternative ways of providing temporary accommodation for people experiencing homelessness.

We next explore the nature and causes of homelessness, followed by a review of the literature on the experiences of households with a history of homelessness living in temporary accommodation and an introduction to the Cambridge local housing context and the modular housing project specifically. We then elaborate our research methods and present evidence regarding the impact of the modular housing on residents’ lived experiences. Our discussion concludes by highlighting key issues that warrant attention of commissioners of similar schemes elsewhere.

## Literature Review

### Understanding Homelessness and Approaches to Providing Housing and Support Services to People Experiencing Homelessness

Defining homelessness remains a challenge because, as Pleace and Herman (2020) argue, defining someone as homeless requires a working definition of what constitutes a home and what does not, as well as overcoming the logistical challenges of accurately counting people experiencing homelessness. This has given rise to using the number of people sleeping rough as a common indicator of the scale of homelessness in most countries (see MHCLG, 2018 for England). This working definition however excludes other forms of ‘hidden homelessness,’ which include people sleeping in cars, tents, on public transport, in night shelters, and those sofa surfing. Homelessness has been linked to both structural constraints and subjective factors (Fitzpatrick, 2005). The former relates to policies, systems, and accepted practices over which households have no control, including unaffordable house prices and thus mortgages pushing households into debt, shrinkage in the provision of social housing, regulatory constraints on releasing land for affordable housing projects, labour precarity (Holmes & Burgess, 2021; Bramley & Fitzpatrick, 2018). The subjective factors relate to personal circumstances and decisions that households make within existing structural constraints that predispose them to homelessness. Examples include, experience of mental health, abuse, family breakdown, strained family relationships, poverty, and exiting from a custodial sentence (Fitzpatrick, 2005; Holmes & Burgess, 2021).

The complexity of homelessness and variations among those who experience it necessitates the provision of both housing and support services to cater to the needs of different households in strategies devised to address the issue (Anderson, 2010). In England as elsewhere, temporary accommodation remains the predominant form of housing provided for people experiencing homelessness (MHCLG, 2021a). Although temporary accommodation is a constellation of different forms of shelter, they have been modelled around what is called the Treatment First or the ‘staircase’ approach (see Annika & Jöhnsson, 2011; Jonhsen & Teixeira, 2012) that withholds independent and permanent housing from people experiencing homelessness until they have demonstrated significant progress in aspects of their lives perceived as predisposing them to homelessness (Johnsen & Teixeira, 2012).

According to Johnsen and Teixeira (2012), this approach is founded on a philosophy which emphasises ‘detoxification’ and ‘sobriety’ before people experiencing homelessness can access independent housing. The proponents of this approach commonly justify its relevance based on practical arguments around the limited supply of affordable housing, especially in high-demand areas, and opportunities for health specialists and service providers, including charities and faith-based organisations, to exercise agency in delivering support to people experiencing homelessness (Padgett et al., 2016; Johnsen & Teixeira, 2012) also draw attention to how, in the UK, the Treatment First approach is compatible with how budgets are split among government departments given the impracticality of transferring cost-savings from one area (e.g., hospital accident and emergency departments) to another (e.g., criminal justice).

### Experiences of Households With a History of Homelessness Living in Temporary Accommodation

The lived experiences of people with a history of homelessness in temporary accommodation are mixed, and often vary based on where they are housed, e.g., in flats, houses, bedsit, bread and breakfast hotels, hostels, women’s refuges, etc. (Mitchell et al., 2004). In terms of advantages, it is widely acknowledged that without temporary accommodation, people threatened with homelessness or living in precarious housing will be sleeping rough and consequently exposed to poor physical and health conditions (Watts et al., 2018). Empirical studies also suggest that support services provided in temporary accommodation positively impact service users. For example, in Mahoney’s (2018) study on discipline and regulation in hostels in Stoke-on-Trent (UK) residents reported that support workers regularly assisted them with activities such as planning to meet short, medium, and long-term action plans, offered counselling, reminded them to make calls to external service providers. Studying the nature of friendships among homeless drug and alcohol users living in three UK hostels, Neale and Brown (2016) observed that considerable ‘social capital’ and ‘recovery capital’ existed within hostels. Their study highlights seven types of friendships (i.e., family-like friends, using friends, childhood friends, online-only friends, drug treatment friends, work friends, and mutual interest friends) formed within hostels go further in helping to overcome the isolation felt by drug and alcohol users in homeless shelters. In addition, they observe that people experiencing homelessness living in hostels particularly valued routine and regular contacts from family-like friends and support workers. Neale and Brown (2016) also studied ‘social and recovery capital’ among homeless hostel residents who used drugs and alcohol. Among other findings, they observed that after moving into hostels, residents experienced positive relations with their families, professional service providers, other hostel residents, friends outside the hostel and with current and ex-partners. Other studies also highlight positive experiences in terms of institutional support for harm reduction, opioid substitution, residential detoxification and rehabilitation, counselling, managing finances, opportunities for education, training, and employment (Neale & Brown, 2016).

Although temporary accommodation provides benefits for people with a history of homelessness, such forms of housing are also criticised for several reasons. For example, a study by Watts et al. (2018) into the nature, purpose, and use of temporary accommodation in Scotland found that bread and breakfast accommodations received the most consistently negative comments from service users for their imposition of rules (e.g., curfews), routines, and lack of facilities for food storage, cooking and laundry. For hostels, residents decried the lack of autonomy and sharing facilities like bathrooms and kitchens with other people. Also, in a survey of 417 homeless people and their children living in temporary accommodation in London and East England, Mitchell et al. (2004) found that 35% agreed that their housing was damp and mouldy, with people living in bedsits, bed and breakfast hotels, and hostels concerned about lack of cooking facilities. Also, some analysts have suggested the fixed location of most temporary accommodation for people experiencing homelessness in cities often remains invisible, and their spatial fixity pre-configures the different geographies (such as of social capital, economic opportunities, influence) they can access outside their accommodation (Jackson, 2015; Šimon et al., 2020).

## The Study Area

The empirical research underlying this paper was conducted in Cambridge – one of the most economically prosperous cities in the UK (Cambridgeshire and Peterborough Independent Economic Review (CPIER), 2018). The population of Cambridge has increased from 123,900 in 2011 to 125,063 in 2020 (Cambridgeshire Insight, 2021), with an age profile of young people aged under 24 constituting 37% of the city’s population (Cambridge City Council, 2019a). Cambridge’s dwelling stock also increased by 16%, from 48,380 to 56,520 between 2011 and 2021 (Cambridgeshire Insight, 2021), although this increase is inadequate for the assessed housing needs in the city.

House prices in Cambridge continue to be among the highest in the country. The average property price in Cambridge as of March 2021 stood at £523,818, as opposed to £382,255 for the East of England and £348,984 for the whole of England (Cambridgeshire Insight, 2021). In 2019, the mean monthly cost of renting a two-bedroom house in Cambridge City and South Cambridgeshire was £1,190 per month and £893 per month respectively (Cambridge City Council, 2019a). Private renting in the city is unaffordable for many Cambridge households (see Cambridge City Council, 2019b) and this has been acknowledged in the Greater Cambridge Housing Strategy 2019 to 2023.

Like most economically prosperous cities in the UK, Cambridge faces the problem of homelessness. Data from the ONS (2020) show that in 2020, for every 1000 households in Cambridge, six were homeless and four were threatened with homelessness. The Autumn 2018 count of rough sleepers identified 27 individuals who slept rough compared to 26 people in 2017 and 40 people in 2016 (Cambridge City Council, 2019b).^1^ Between 2013 and 2019, 24 homeless people died per million of Cambridge’s population (ONS, 2020), and the City Council’s Homelessness and Rough Sleeping Review cites the loss of a privately rented home and the unwillingness of friends and family members to provide accommodation as the two most common reasons why people lost their last settled homes, with both factors accounting for almost 50% of all seven causes of homelessness identified in the city (Cambridge City Council, 2019b).

In recognition of its homelessness challenge, the City Council has recently initiated several interventions which include, but are not limited to, providing discrete temporary accommodation to single households and families experiencing homelessness, resourcing effective housing services, and partnering with local interest groups and stakeholders to explore collaborative ways of tackling homelessness and rough sleeping (Cambridge City Council, 2019a). Significantly, the City Council has also recently published its Homelessness and Rough Sleeping Strategy 2021–2026 in which it sets out six priority areas of tackling homelessness. Notably, Priority Six ‘Breaking the cycle of chronic and repeat street homelessness and rough sleeping’ commits to:

‘… expand the provision of modular homes and explore innovative ways of providing accommodation to prevent and relieve single homelessness’ (Cambridge City Council, 2021:11).

It is within this context that the modular housing project for tackling homelessness was set up to start in 2019.

### The Modular Homes Project

Our case study is the city of Cambridge’s first modular homes project, which also happens to be the first project of its kind in England. It was conceived as a collaboration between Cambridge City Council and three local organisations: *Jimmy’s Cambridge*, a local homelessness charity that provides emergency help, support, and accommodation for people experiencing homelessness in Cambridge; *Allia*, a social enterprise that supports social and environmental impact organisations with space, support, and access to capital; and *New Meaning Foundation*, a social enterprise which specialises in individual attitude development, ethical construction, and social action (Burgess et al., 2020a, b). The shared goal of this project was to provide individuals experiencing homelessness with housing complemented by wrap-around support services designed to help them overcome substance use and other life challenges (ibid.).

The project comprises six self-contained units, each measuring 25 square metres. The units were produced using modular construction techniques (Burgess et al., 2020b), which entails the design, production, and assembly of components of construction projects in a factory before they are installed on site (Burgess et al., 2020a, b). The ‘meanwhile site’ leased for the project is owned by a church in Cambridge that has no immediate use for the land but has long term plans for its redevelopment. Accordingly, planning permission for the project was granted for three years from mid-October 2019, on the condition that the project would be a transitional phase to help tackle homelessness in the city. However, the units could be moved to a new site when the planning permission expires and the church is ready to redevelop the land.

**Fig. 1:**
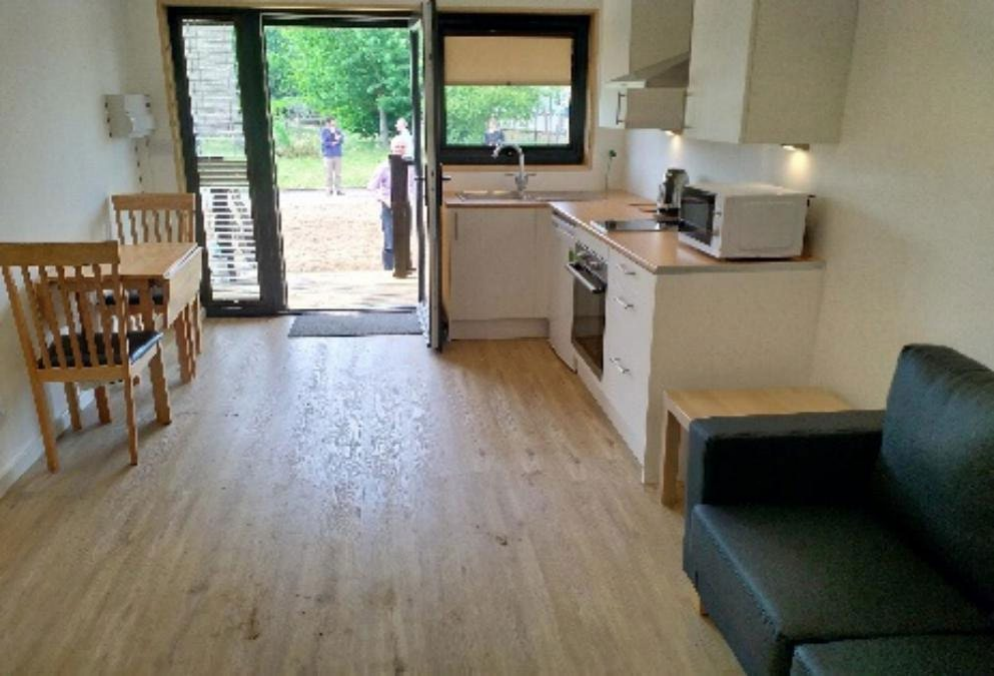
Interior view of the modular homes with white goods and furnishings. (Source: Authors’ site visit (June 2020))

**Fig. 2:**
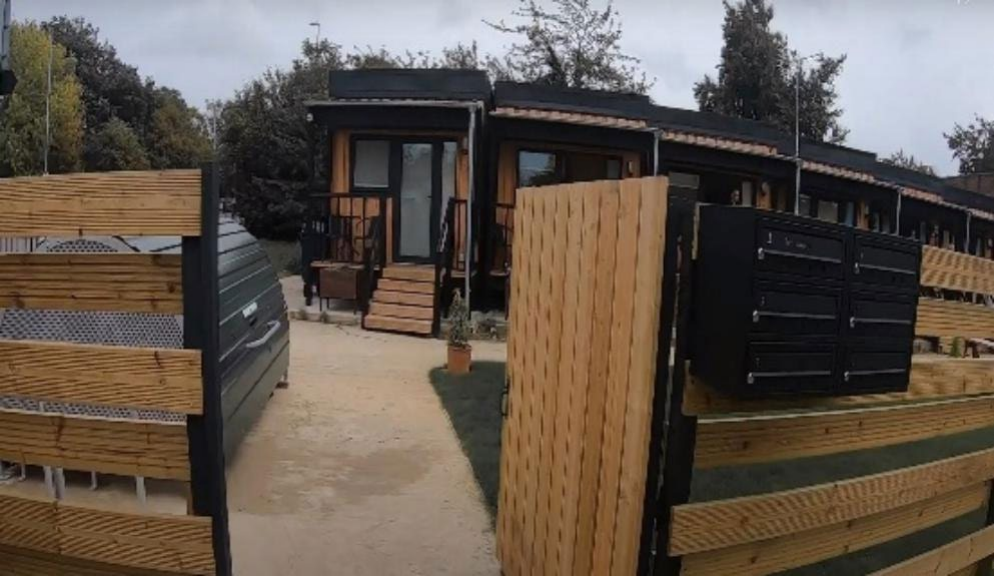
Exterior view of the modular homes showing frontage, letterboxes, communal garden and bicycle storage

Several local stakeholders offered in-kind contributions towards different aspects of the project, including land acquisition, legal services, planning consultancy, manufacturing and storage of the units, groundworks and on-site installation, engineering, landscape design and finishing, and fit-out such as upholstery, lighting, kitchen sink and countertops, and decorated walls. Each of the units has a private bathroom, and is fully furnished with living and bedroom furniture, white goods, crockery, and a flat-screen wall-mounted television. Each unit has its own front door with a porch area, and there is a communal garden and a bicycle shed. The site is fenced with a low, wooden fence and the shared frontage is provided with individual letterboxes (Figs. 1 and 2).

The selection of residents to occupy the modular homes was made with input from different stakeholders of the local ecosystem of homeless service providers and local councils. First, the local homelessness charity, Jimmy’s Cambridge, drew on its database of people accessing homelessness support to shortlist 13 potential residents. This list was presented to both the City Council and the Street Outreach Team – Cambridge (Change Grow Live) and reduced to six shortlisted candidates. Initially, the short list included one female candidate, but prior to the moving-in date, she was withdrawn from the shortlist due to a significant change in personal circumstances, and her place was offered to another male candidate. Overall, the criteria for selecting the residents were informed by a combination of individual housing needs and the perceived level of support that residents would require to help them move on into permanent housing. The level of support needs was deemed to be medium to low, excluding for example people actively using drugs and people with a diagnosis of a mental health condition. Being the first of its kind in Cambridge, the project stakeholders also felt the need to strike a balance between people who were considered as ‘high risk’ and would need greater support to live in the modular homes and those with a higher prospect of progressing to independent living with limited support from Jimmy’s (Burgess et al., 2020a, b). This setup was further supported by choosing a resident with low support needs and stable circumstances to act as a warden, a condition built into the planning permission. The warden’s role is to liaise between the residents and the team of floating support workers who were required to be on site for up to six hours during each working visit and contactable 24 h a day, seven days a week. The COVID-19 pandemic delayed both the initial date for installation of the modular units and the move-in dates. However, in July 2020, the first group of six residents moved in.

## Methods

Our study is exploratory and not intended as an evaluation of the effectiveness of modular homes in tackling homelessness. Our interest lies in bringing together what can be learned from the project based on the lived experience of the residents to make available to groups and organisations contemplating similar schemes elsewhere. Our exploratory approach is important as these projects are very new in England (and indeed around the world), and many more are being planned for implementation across the country (Karampour & Burgess, 2022).

Consequently, we adopted a qualitative method grounded in an interpretivist ontology to allow residents to subjectively reflect on their individual lived experiences before and after moving into the modular homes. Persaud et al. (2010) note that lived experience is a key viewpoint in qualitative research, relevant for understanding a person’s unique perspectives on their daily experiences and how that reflects their social and cultural worlds. As COVID-19 lockdown restrictions in England were still in place when the residents first moved into the modular homes, video interviews over Zoom were used for data collection.

We interviewed a total of 18 participants: six residents, five project stakeholders, two in-kind and cash donors, and three support workers. The first set of interviews was with residents, and took place in four rounds, the first of which was conducted briefly before residents moved in. These interviews provided us with a baseline understanding of the expectations people had about the modular homes. We conducted the second set of interviews a week into their residency to continue learning about their past experiences, including how and why they became homeless. We also used the interviews to ascertain residents’ initial impressions of the modular homes and their expectations for the next two years of their stay. The third round of interviews was conducted six months into their residency to find out about their experience, what difficulties they were facing and how they addressed them. We conducted a final round of interviews in July 2021 (12 months after move-in) to ascertain residents’ subjective evaluation of their progress and future housing trajectory. On each interview day, a support worker from Jimmy’s Cambridge visited the residents and passed around an iPad for the interviews. Each interview session typically lasted between 40 and 60 min and was recorded with residents’ permission. Our interviews with project stakeholders aimed to gauge their expectations for the project and what would count as success. They included Jimmy’s senior management, a representative of the church that offered the land, the project champion – who was instrumental in the project financing, land acquisition, planning application, partnership formation, and furnishing of the units, and officials from the Cambridge city council. For project donors, we designed our interviews to elucidate their motivation for contributing to the project and what would count as success, while in interviews with support workers we sought to corroborate the accounts shared by residents and to obtain support workers’ perspectives regarding both the challenges each resident was facing and the progress they believed had been made so far. Ethical requirements were adhered to.^2^ Finally, we transcribed the recorded interviews using the audio transcription software Otter.ai. To ensure trustworthiness, we provided interviewees with the verbatim transcriptions to confirm that they captured their views accurately.

In analysing the transcripts, we used the common thematic areas highlighted when analysing the impact of homelessness interventions such as Housing First Pilots (see MHCLG, 2021b). These comprised improvements in areas of livelihood such as: (1) drug and alcohol use; (2) money management skills; (3) employment, skills development, and readiness for work; (4) relationship management; (5) sense of community, safety, and security; and (6) intended housing trajectory. Using these livelihood categories, we examined how residents described the changes they had experienced since moving into the modular homes and the specific evidence they adduced to support their claims. We present relevant extracts from the transcript that illuminated specific challenges that residents’ previously experienced and the positive impacts that living in the modular homes had on them as evidence of impact. Approval for the use of any quotes and interpretations drawn from them was granted by the residents and the homelessness charity. All interviewees were anonymised.

## Background: Demographic Information and Abbreviated Life Histories of the Residents

All six residents living in the modular homes are single men aged between their late twenties to early sixties; all have educational attainments below a university degree. Two have children who do not live with them. This demographic profile reinforces recent findings from surveys in England and Scotland that the odds of experiencing homelessness are higher among single adult men and single parents (Bramley & Fitzpatrick, 2018). Before experiencing homelessness, all residents were engaged in some form of economic activity, with previous jobs including painting and decorating, cabinet and furniture making, construction labour, and retail assistant.

Also, consistent with the literature (Fitzpatrick et al., 2021), the initial trigger for moving out of stable accommodation varied markedly among the residents. The reasons given for becoming homeless included disputes with close family members, loss of employment, eviction following a landlord’s decision to sell the property, death of a partner who was responsible for paying the rent, and loss of close family, housing, and all personal assets in a fire.

The length of time that residents had been sleeping rough or moving in and out of temporary accommodation varied. For example, one resident had been homeless for over 20 years, another had been in and out of temporary accommodation for over 10 years, and others had served short custodial sentences. The residents shared a common struggle with drug and alcohol use, which some said helped them to cope with anxiety and challenging life experiences.

## Results: Impacts of Living in the Modular Homes

### Drug and Alcohol Use

Almost all the residents struggled with drugs and/or alcohol use, which negatively impacted multiple aspects of their lives. Such negative impacts included but were not limited to difficulties in maintaining good mental and physical health, finding or keeping a job, sustaining a tenancy, making sound financial decisions, maintaining relationships, regular encounters with the criminal justice system, and difficulties in accessing medical assistance. Reflecting on their experiences over the 12 months, residents indicated how living in the modular homes and receiving support services helped them make positive behavioural changes in dealing with their drug and alcohol use. One resident remarked:

Living here, oh everything is good! I can’t say anything bad. It’s got me off drugs, got a roof over my head, it’s got me back to work…can’t say anything bad really. I’ve stopped using drugs altogether. There’s nothing bad about the place. Everything’s positive.

Our findings also suggested that, in addition to residents’ individual determination to make improvements while living in the self-contained modular homes, a review of the conditions in the tenancy agreements residents signed may have also contributed to the positive impacts as their license agreement explicitly forbids the use of illegal drugs and alcohol in the units: ‘The licensee will not have, use or supply (or allow visitors to have, use or supply) illegal drugs in The House’ (Paragraph 8 of the License Agreement). These conditions notwithstanding, it is worth noting that all residents volunteered to live in these modular homes and, before moving in, pre-tenancy meetings were held by the homelessness charity to provide an opportunity for the residents to discuss and be aware of the conditions for their continued stay in the modular homes. Residents were also advised that random checks would be conducted by support workers during their stay to ensure that they were not breaking this condition of their residency. One resident in particular actively demanded regular drug tests that helped him keep himself accountable. The majority of residents throughout our study adhered to these conditions while living in the units. One, however, was evicted, based partly on his drug use leading to behaviours characterised as ‘seriously disruptive and violent’ under Paragraph 10.4, Grounds for Ending License Agreement (Jimmy’s Cambridge, 2020, p. 8).

### Money Management Skills

Before moving into the modular homes, some residents faced difficulties in managing their finances and as a result they were unable to regularly pay rent and other bills promptly. Living in the modular homes came with financial obligations, and the License Agreement stipulated that residents were responsible for weekly charges, comprising housing benefit claimed from the council, any ineligible personal charge, and the licensee’s rent. Failure to honour these commitments constituted grounds for ending the tenancy under Paragraph 10.2(i) of the License Agreement (2020). We found that offering residents advice on budgeting and managing their money as part of the wrap-around services provided helped some of them to improve their money management skills, with two-thirds confirming that they were paying their rent and service charges promptly. To help the remaining one third who had missed their weekly rent payment at some point, additional support and flexible payment terms have been extended to help them sustain their tenancy. Residents were aware that defaulting on rent payment has implications for their tenancies – ‘a problem’ and hence there is a strong incentive to honour their commitment whilst also developing the skills and discipline around financial management. As one noted: “I have been paying my rent on time and there is no problem.”

For other residents, the improvement in money management is best illustrated when faced with a choice over how to use windfall income, as one described:

I’ve managed to get my sick pay, which was backdated. It wasn’t a lot but enough…. I had a choice to buy a motorbike, which I really did want, or to buy tools because I need them to try and get myself back into some kind of work or whatever. So, I decided, ‘Right, buy the tools.’ I made a commitment, bought the tools. I’ve bought all of the tools.

As can be seen, the improvements residents are making in their money management skills extend beyond just honouring their financial commitments as part of living in the modular homes effectively to preparing for manging income from employment, necessary to support independent living.

### Employment, Skills Development, and Readiness for Work

Our initial interviews with the residents immediately after they moved indicated a reluctance in terms of finding work. This may have been partly attributable to the need for them to first settle into their new homes before focusing on finding employment. However, one year into their residency, our evidence suggests that residents are keen to (return to) work. As we noted earlier, not all the residents were jobless before experiencing homelessness, and our findings show that among those previously employed some are planning to return to their previous line of work. For example, a resident who sold newspapers but had stopped during the COVID-19 lockdown, resumed work following the easing of lockdown restrictions. Another resident, who was a skilled cabinet and furniture maker but had not worked for a long time, seemed keen to return to his trade. He observed:

The job I am familiar with is how to make cabinets and furniture and decorate houses. I want to work till retirement. I’m returning to my passion. I’ve got a goal; I’ve got a plan. I can’t believe I’m saying that but yeah…it’s amazing really.

Not only motivated to return to work, this resident sees a return to work as part of his plan towards the realisation of his goal of being able to retire. For those residents who were not previously employed, we found that with the assistance of the homeless charity, some were learning new skills, including through specialised training (e.g., in construction, barbering). The enthusiasm and commitments that residents had demonstrated in terms of their readiness for work was corroborated by the support workers who had worked closely with them to, among other things, help them register for and take tests required for starting work and training courses, and offering suggestions for the purchase of relevant work equipment:

We’ve got one individual who has got a CSCS^3^ test… and he starts a new job within the next week. He’s already been offered the job; he’s just got to do the CSCS test for working on a building site before he goes into full-time work. I think six months ago when you spoke to him about returning to work, that wasn’t even an option. We’ve got another resident who’s just going to start college in September to do barbering. – Support worker, Jimmy’s

Importantly, beyond providing shelter for the residents, the modular homes have also given them a residential address that can be used in completing employment-related forms and accessing various welfare benefits.

### Relationship Management

From their backgrounds, we learnt that the breakdown of social relations was one of the pathways leading some residents into homeless. For this group in particular there was a strong desire to restore broken relationships and to reconnect with family members once they secured decent and stable accommodation. It is worth noting here that prolonged homelessness and destructive behaviours such as drug use can make it difficult for people experiencing homelessness to reconnect with family members and restore familial relations. Another reason for some people to choose not to connect is feeling shame. One resident, whose son had been taken into foster care because of his lack of stable accommodation, intimated that living in the modular homes for the past 12 months has given him grounds to apply for custody of the child. He remarked that:

I am doing everything I can to get back my child. That place [referring to foster care] is not good for him… So, I am staying clean, and I hope that in no time I can get my rented place and get back my child. … I love that boy, I don’t want him to be put in foster care. […] Me and the social services don’t really get on very well. So, luckily for me, I’ve got [support worker] on my side who can speak up for me… I ask him to do my speaking for me if I need to speak to them.

Two points are noteworthy here. The first is the allusion to ‘staying clean’ – a reference to not using illegal drugs while living in the modular homes and not continuously disengaging with the support services offered for tackling addiction issues – which for this resident signals an ability to remain drug free and thus sustain his tenancy. The second is that there is evidence of a healthy, trusting relationship between residents and support workers, such that one support worker is willing to testify in a custody hearing about the progress that a resident has made in staying sober and maintaining a tenancy.

Another resident said living in the modular home has helped him to tackle his alcohol use and to reconnect with his daughter for the first time in over 20 years:

Before moving here, I barely spoke to my daughter. Now I talk to my daughter every week, twice a week. Living here has allowed me to make some improvements. I’ve been clean for 14 months. She’s coming to see me here for my birthday in July.

Based on the above it is plausible to argue that his modular home – in terms of size, furnishing, exclusive access to front doors, security arrangements – offers a congenial atmosphere for him to host his daughter.

These responses indicate that there is a strong connection between having comfortable, stable housing, and receiving adequate support and reconnecting with family members. Living in the modular homes offers more than shelter as it can, on the one hand, inspire a sense of self confidence overcoming shame and eventually even engender pride among residents to invite close relatives to their new home, and at the same time provide evidence to demonstrate they are able to lead a responsible life.

### Sense of Community, Safety, and Security

Our interviews showed that a sense of community has developed among the residents since moving into the modular homes. This is reflected in a combination of external, communal, and individual initiatives, including being invited to the local church’s Christmas celebration – a gesture the residents described as creating a ‘good feeling.’

The bespoke nature of the units has also attracted curious passers-by to occasionally stop and engage residents in brief conversations. Whilst some residents view such encounters as intrusive, they can be considered as a means of fostering positive social interaction between the residents and the public, and of raising awareness about the modular housing project. Internally, residents get along well with each other and interact mainly through (un)planned conversations across porches or over tea in the shared garden on the premises. They all expressed keen interest to support regular communal meals and outdoor socialising events during the summer months. Illustrating the growing sense of trust between some of the residents, borne out of the sense of community, one described how another resident had confided in him regarding a medical condition and imminent surgery:

Yeah, everyone has been good, it’s only [fellow resident] who told me he was suffering from terrible back pain some time ago, but he’s going to go for surgery to fix that soon.

The shared sense of community also feeds into the sense of safety and security felt by all residents. Our first round of interviews revealed that residents had experienced insecurity in previous accommodation because of the behaviour of other tenants. One resident described how he had left a shared house after being threatened by a fellow resident and gone back to sleeping rough, despite the dangers. However, a year into their residency in the modular homes, residents were feeling a greater sense of safety and security:

Well, living here is safe for me and I like it. I’ve got my own place, I can stay locked in to prevent any trouble from others, but the guys are generally nice so there’s no trouble at all. We’ve got cameras around here and they help to keep us safe.

The sense of safety connects first, to the fact that the units are self-contained and fully furnished prevents low level disagreements over shared items and offers protection against outsiders; second, the sense of community that the residents have developed can be relied upon to ward off potential intruders on site, reinforced by the security cameras installed on the premises; and third, the modular homes offered not only a shelter but one’s own space and ‘own front door,’ which allows a certain level of independence and also a sense of personal ownership that extends to the ability to make decisions for oneself. We heard repeatedly from residents that having one’s front door is very much a benefit of the modular homes versus hostel or other kinds of shared housing offerings.

### Future Housing Aspirations

We found mixed future housing aspirations among the six residents, underpinned by different motivations. Some aspire to move into permanent accommodation for familial reasons:

Yeah, they will not let me have my boy until I am clean and have my flat, so I have to find my own place. [….] My friend was in a similar situation, so he rented a flat and applied to take back his child and he was successful, though he was still a heavy drinker.

The extent that this resident’s desire to move into permanent housing is inspired by a sense of readiness or desperation to obtain custody of his child is unclear. This is important because according to a support worker the time and degree of support residents require to feel confident about moving into independent housing varies, for example one described a young resident as:

[He] is young and doesn’t have problems with drugs or anything. However, he needs a lot more work in terms of money management, mental health and wrap-around support. His mood gets quite low, and his anxiety seems to have been worsened by COVID. He is not ready to move on. *–* Support worker, Jimmy’s

It is critical to offer people experiencing homelessness the maximum amount of appropriate support for as long as is practically possible, especially given that individuals may over-estimate their progress in their bid for independence. This is important in helping balance a strong desire and motivation to ‘move on’ with the need to avoid making a premature decision to move into permanent independent housing before being fully prepared to do so.

Other residents saw moving out of the modular home into permanent accommodation as a pathway for (re)integrating into society and leading a ‘normal life.’ It is however important to note that there was a case where support workers confirmed the readiness of one resident to move into permanent independent housing:

[He] is moving out imminently. We’ve helped him get a Band A, so he’s looking forward to getting his property. But it has just been slow with the move-on property due to COVID. – Support worker, Jimmy’s

In contrast, some residents do not currently want to move out of the modular homes if the units will be relocated to sites within Cambridge, for example, one resident revealed a sense of place attachment, but also attachment to the units and the bespoke configuration of the support:

I am not looking forward to being told where I am going to be put next after here. I hope the three-year limit will extend to five years. It will be difficult for me to move out but if the next location is not outside Cambridge, then that will be fine.

Altogether, the varied housing trajectory aspirations show that people experiencing homelessness can be helped to find ‘permanent’ housing in different ways that should be recognized by stakeholders involved in tackling the problem of homelessness.

## Discussion

Notwithstanding the positive outcomes this project has delivered to residents, there are important issues to be considered by key stakeholders and policymakers elsewhere considering modular homes as alternative temporary accommodation for people experiencing homelessness including the impact the project has had beyond the individual resident’s outcomes.

Firstly, the reliance on ‘meanwhile’ sites for these projects may present a significant risk given the difficulties in finding land in suitable locations. While our case study project is sited in a central location in Cambridge that offered easy access to key amenities (e.g., electricity, sewage, internet, water, transport, recreational facilities) deemed critical in residential location decision-making (see Balta & Öztürk 2021), future schemes elsewhere may not enjoy similar locational advantages. Indeed, a nationwide survey of modular homes indicated that some container and modular homes for people experiencing homelessness lacked additional onsite facilities such as play areas for children, or communal and open green spaces (Karampour & Burgess, 2022). Project promoters should carefully consider the location of similar projects since it will have implications for the wellbeing of residents (cf. Mingoya, 2015). We strongly advocate further research examining the contribution of project location to improve residents’ outcomes, as well as, considering the early stage of our understanding of modular homes generally, whether the reliance on ‘meanwhile’ land can be sustained and what alternatives may be available.

In our case study, ‘meanwhile land’ was used to offer residents a limited tenancy based on a 3-year lease. At the time of writing, the landowner had neither requested extended planning permission for the land nor indicated immediate permanent development plans for after the current lease expires. Thus, it is possible that the modular homes will be able to remain on the site if the planning permission is renewed. However, the modular homes are intended to provide temporary accommodation in the expectation that after three years the first cohort of residents will have made progress towards independent housing making them available to accommodate a new cohort if the site remains available. However, not all the residents in our case study demonstrated readiness to move into independent housing. A strong need for clarity appears in terms of whether residents can continue in the modular homes after the expiration of their three year lease if they are insufficiently prepared to move on. This is particularly important given that the planning permission was issued for temporary accommodation, requiring tenants to move on even if the site remains usable. Clarity on this is important is necessary because if residents must leave at the end of their tenancy period regardless of their readiness for independent living, any gains they may have made during the period of supported living could be reversed. Thus, consistency – at least in terms of communication – is vital.

Third, since some residents expressed a desire to continue living in the modular homes (after the three years and even after the relocation of the units), it may be possible to include these (or other) self-contained modular homes within the available move-on and long-term housing options. Indeed, one resident reported during the interviews that he felt lost and struggled to cope when placed in a flat with larger spaces. Thus, the conventional idea of independent housing cannot be conceived as a “one size fits all” solution, which aligns closely to recent discussions about what ‘a dwelling’ must look like in the radical housing literature (see Lancione, 2020). Cambridge City Council includes modular homes in the options of move-on homes for people experiencing homelessness, supporting our findings that suggest, for some, it is easier to sustain a long-term tenancy in a modular home with their ‘own front door’ than in other, often shared, housing options.

Fourth, while the support services offered within modular homes seem to mirror the Treatment First approach, these modular homes differ significantly from the conventional ‘brick-and-mortar’ on-site temporary accommodations for people experiencing homelessness in terms of the cheaper cost of units, minimal on-site variations, shorter delivery time, environmental-friendliness, and the potential to re-site units (e.g., Ehwi et al., 2022). These advantages make the use of modular homes as temporary accommodation crucial, especially as the UK housing market is characterised in part by a stagnant supply of social housing between 2008 and 2018 (ONS, 2019), rising house prices (Sissons & Houston, 2019) and a growing number of people threatened with homelessness (Fitzpatrick et al., 2021), all contributing to longer waiting times to access long-term housing.

In our study project, residents are able to re-decorate and reorganize the interior of their units to better reflect their tastes and preferences, and are permitted to host guests such as family members. However, they cannot alter other structural components of their units, including but not limited to design, the size, construction materials. This should not detract from the opportunity of home-making the modular homes provide residents, not offered to any comparable degree by other forms of temporary accommodation, like bed and breakfast hotels, day centers, and hostels (Jackson, 2015).

Also unlike other forms of temporary housing, modular homes offer residents the opportunity to experience the city environment and navigate its different geographies (social capital, economic opportunities, and even risks) in different ways, even if the project is relocated to different sites across a city (Jackson, 2015; Šimon et al., 2020). At the same time, this possibility provides an opportunity to influence how the wider society views homelessness and approaches those who experience it. Indeed, as we found, the siting of the scheme close to an already existing local community created opportunities for community members to interact with the residents, providing opportunities for learning and potentially challenging derogatory stereotypes of people experiencing homelessness. A visit by the Duke and Duchess of Cambridge to our study project illustrates the visibility such schemes enjoy (see Leishman 2022; ITV News, 2022)^4^.

It also instructive to highlight that the impact the modular homes and their residents are having beyond enhancing public awareness of homelessness in profoundly impacting the type of temporary accommodation offered to people experiencing homelessness. As noted above, the Cambridge City Council incorporates the provision of modular homes in its 2021–2026 Homelessness and Rough Sleeping Strategy as an alternative form of temporary accommodation, and another 22 local authorities in England have followed suit (Karampour & Burgess, 2022). Notably, the Department of Levelling Up, Communities, and Local Government (DLUHC)^5^ highlights its support for research on new schemes for tackling homelessness including modular homes (DLUHC, 2022). The implication is that government funding will likely be made available to support research related to this new form of temporary accommodation. Nevertheless, questions remain about how these new schemes tackle the structural factors contributing to and perpetuating homelessness (Fitzpatrick, 2005) or whether they are part of an attempt to simply reduce the statistics on rough sleeping across cities, and possibly attenuate or distract from the strong argument for both government and local authorities to provide more socially affordable housing for households priced out of the housing market and facing precarious housing conditions.

Finally, it should be borne in mind that that this is a pilot project and the residents selected to occupy the units were drawn from individuals who had at some point engaged with Jimmy’s (the homeless charity). We cannot claim that similar outcomes will be achieved for new schemes that target people with more complex support needs and who have not otherwise sought assistance. Our sample is dominated by single adults with less complex needs (see Bramley & Fitzpatrick 2018). Thus, it will be helpful to learn how different cohorts of residents will fare in the modular homes, having regard to personal characteristics (e.g., gender, household type, ethnicity, etc.), different housing designs (e.g., multiple storeys) and levels of support needs (e.g., moderate to complex). It is also unclear whether there is an optimum number of modular homes per site to sustain the positive outcomes realised from this case study. Furthermore, a robust evaluation comparing modular homes to other more common forms of temporary accommodation might yield a better understanding of the key factors leading to the positive outcomes documented.

## Conclusion

Our findings document how homelessness is being tackled through a novel approach that involves the use of self-contained modular homes and wrap-around support services in the city of Cambridge. Based on longitudinal interviews with residents, we found that residents experienced positive outcomes across a variety of dimensions: improvements in drug, alcohol, and money management skills, readiness for employment and skills development, improved social relations, a burgeoning sense of community, and a sense of personal safety and security. However, the future housing trajectories of the residents varied, driven by different motivations that highlighted the need to create room for innovative move-on housing arrangements to cater for individual requirements.

As this new approach to tackling homelessness continues to gain support across the UK (see Karampour & Burgess, 2022) and beyond, more empirical research is needed to understand issues regarding the design and implementation of these schemes. Also, further studies involving larger and more diverse (in terms of gender, needs requirements, ethnicity, etc.) cohorts of residents living in different kinds (e.g., number of units on site) of modular housing and receiving varying levels of support, is needed to better capture the key factors that help deliver positive outcomes. We are aware of potential debates around whether these modular homes are merely token interventions by city leaders to shift public attention away from the shortage of more affordable social housing or present a ‘convenient partnership’ to allow local politicians to reduce the number of homeless rough sleepers in their cities. However, we argue that our case study demonstrates that providing people experiencing homelessness with their ‘own front door’ in conjunction with ‘wrap-around’ social services can allow them to readjust to fruitful participation in the wider society, including with their own families, and that this option should therefore be included in the array of temporary accommodation and social services offered to people experiencing homelessness worldwide.

## Data Availability

The datasets generated during the current study are not publicly available due to sensitivity and confidentiality of data but are available from the corresponding author on reasonable request.

## Footnotes

1 We know from conversations with stakeholders and research during the first phase of Covid (including in emergency Covid Hotels) that this number is a drastic underestimation, and the number of people sleeping rough in Cambridge on any given night is likely to be closer to 100 (i.e., three times as high as the official count).

2 These included explaining the purpose of the research, seeking participants’ consent, completing Disclosure and Barring Service (DBS) checks, and outlining protocols to guarantee participants’ anonymity.

3 CSCS stands for Construction Skills Certification Scheme. It is a test designed to give individuals working in the construction industry the requisite knowledge in the areas of health, safety, and environment, so that they can identify hazards on site and take preventive steps (https://cscstest.org.uk/).

4 ITV is a UK-based television station.

5 That is the government’s acronym in use. https://www.gov.uk/government/organisations/department-for-levelling-up-housing-and-communities.
